# SCD1 Inhibition Causes Cancer Cell Death by Depleting Mono-Unsaturated Fatty Acids

**DOI:** 10.1371/journal.pone.0033823

**Published:** 2012-03-22

**Authors:** Paul Mason, Beirong Liang, Lingyun Li, Trisha Fremgen, Erin Murphy, Angela Quinn, Stephen L. Madden, Hans-Peter Biemann, Bing Wang, Aharon Cohen, Svetlana Komarnitsky, Kate Jancsics, Brad Hirth, Christopher G. F. Cooper, Edward Lee, Sean Wilson, Roy Krumbholz, Steven Schmid, Yibin Xiang, Michael Booker, James Lillie, Kara Carter

**Affiliations:** Genzyme Corporation, Waltham, Massachusetts, United States of America; Enzo Life Sciences, Inc., United States of America

## Abstract

Increased metabolism is a requirement for tumor cell proliferation. To understand the dependence of tumor cells on fatty acid metabolism, we evaluated various nodes of the fatty acid synthesis pathway. Using RNAi we have demonstrated that depletion of fatty-acid synthesis pathway enzymes SCD1, FASN, or ACC1 in HCT116 colon cancer cells results in cytotoxicity that is reversible by addition of exogenous fatty acids. This conditional phenotype is most pronounced when SCD1 is depleted. We used this fatty-acid rescue strategy to characterize several small-molecule inhibitors of fatty acid synthesis, including identification of TOFA as a potent SCD1 inhibitor, representing a previously undescribed activity for this compound. Reference FASN and ACC inhibitors show cytotoxicity that is less pronounced than that of TOFA, and fatty-acid rescue profiles consistent with their proposed enzyme targets. Two reference SCD1 inhibitors show low-nanomolar cytotoxicity that is offset by at least two orders of magnitude by exogenous oleate. One of these inhibitors slows growth of HCT116 xenograft tumors. Our data outline an effective strategy for interrogation of on-mechanism potency and pathway-node-specificity of fatty acid synthesis inhibitors, establish an unambiguous link between fatty acid synthesis and cancer cell survival, and point toward SCD1 as a key target in this pathway.

## Introduction

The fatty-acid content of cells in the body is derived from the diet and from *de novo* synthesis. Rapidly-proliferating cancer cells often have a robust program of fatty acid synthesis accompanied by high-level expression of associated genes such as fatty-acid synthase [1]. Because of its relative abundance in cancer cells, fatty-acid synthase has been pursued as an oncology target [2]. However, it is unclear whether fatty-acid synthase represents the rate-limiting component in the fatty-acid synthesis pathway.

Long-chain fatty acids are critical for the rapid membrane synthesis requirement in vigorously-growing cells and play key roles in various signaling schemes [3]. Additionally, a suitable balance of chain-lengths and degree of saturation is critical for maintenance of membrane fluidity and curvature [4]. It has been reported that inhibition of various steps in the fatty-acid synthesis pathway causes inhibition of cancer cell growth, either because of deficiency in downstream fatty acids *per se*, or because of buildup of toxic pathway intermediates such as malonyl-CoA, or both [5].

Using a combination of siRNA and small molecule inhibitors, coupled with a “fatty acid complementation” strategy, we have identified stearoyl-coA desaturase 1 as an enzyme in the fatty acid synthesis pathway that is essential for cancer cell viability. The “complementation” strategy allowed characterization of both SCD1 as well as the specificity of various fatty-acid synthesis inhibitors, and clarifies the mechanism by which SCD1 inhibition restricts cancer cell proliferation. Our data outline an unambiguous link between fatty acid synthesis and cancer cell survival.

## Results

### Cancer cells are sensitive to loss of SCD1 function

**Figure 1:**
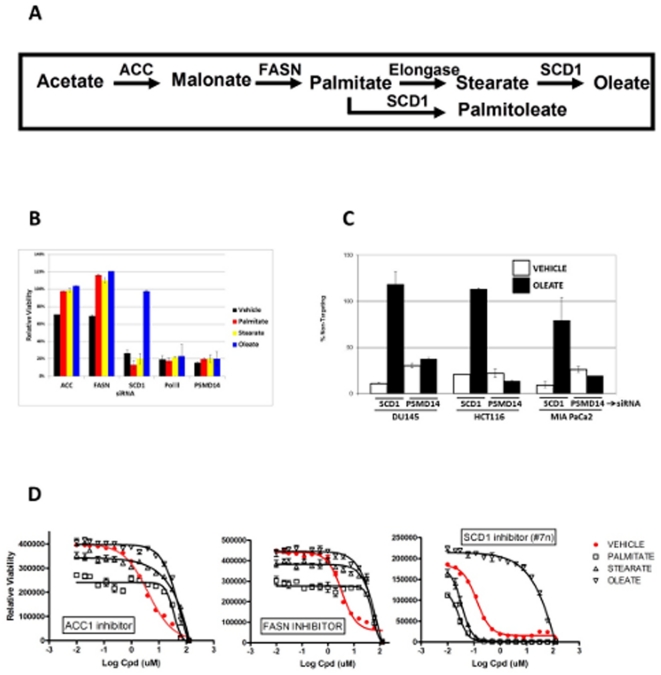
SCD1 is a fatty acid synthesis pathway node critical for cancer cell survival. **A** *de novo* synthesis of mono-unsaturated fatty acids. **B** HCT116 colon cancer cells (ATCC) cultured in RPMI-1640 (Cambrex) containing 2%FBS plated at a density of 4000 cells per well in 100 ul media in 96-well plates were transfected with siRNA pools (Dharmacon, 50 nM) targeting three fatty-acid-synthesis pathway nodes,or two unrelated survival genes, using Lipofectamine 2000 (Invitrogen). 16 hours after transfection, cells were treated with 25 uM fatty acids (Sigma, 100× stocks dissolved in 10%MeOH/0.9%BSA/PBS) as indicated, and viability was determined 72 hours after transfection (Cell Titer Glo, Promega). Results are expressed as percent viability versus cells transfected with a non-targeting control siRNA (designated 100% viability) treated with the same fatty acid. **C** DU145 prostate cancer cells, HCT116 colon cancer cells, and MIA PaCa2 pancreatic cancer cells (ATCC) cultured in RPMI-1640 containing 2% FBS were treated with single siRNAs targeting SCD1 or PSMD14 (Dharmacon, 25 nM), followed 16 hours later by treatment with oleate as indicated. Viability was determined 72 hours after transfection. Results are expressed as percent viability versus cells transfected with a non-targeting control siRNA (designated 100% viability) treated with the same fatty acid. **D** HCT116 colon cancer cells plated at a density of 1000 cells per well in 25 ul media in 384-well plates were treated with small-molecule inhibitors of ACC1 (CP640186, Pfizer), FASN (#10v, Merck), or SCD1 (#7n, Abbott), in media containing fatty acids as indicated, 72 hours prior to viability determination. Inibitors were synthesized at Genzyme (Waltham, MA).

To examine the effect of interruption of fatty acid synthesis on cancer cell viability, we used siRNA pools to deplete three nodes in the pathway for synthesis of long-chain fatty acids (Figure 1A). As shown in Figure 1B, depletion of acetyl-CoA carboxylase (ACC1), fatty acid synthase (FASN), or stearoyl-CoA desaturase (SCD1) results in decreased viability (or cellular metabolism) (as measured by cellular ATP levels using Cell-Titer Glo) in HCT116 colon cancer cells, by 30%, 30%, and 70%, respectively, versus a non-targeting siRNA control, which was designated as 100% viability In each case, mRNA knockdown was determined by real-time RT-PCR to be approximately 80% (not shown). We reasoned that if this cytotoxicity is truly gene-linked and on-target, and if interruption of the fatty acid synthesis pathway results in reduced cell viability owing to a deficiency in downstream fatty acids as opposed to buildup of toxic pathway intermediates, then cytotoxicity caused by depletion of various pathway nodes should be rescuable, or preventable, by addition of exogenous fatty acids downstream of that node. As shown in Figure 1B, ACC1 depletion and FASN depletion are rescuable by palmitate, stearate, and oleate, all of which are downstream of both ACC1 and FASN. SCD1 depletion is not rescuable by palmitate or stearate (which are upstream of SCD1), but SCD1 depletion is significantly rescued by oleate (which is downstream of SCD1). Reduced cell viability caused by depletion of two essential genes of unrelated mechanism, PSMD14 and RNA polymerase II (Pol II) is not rescued by any of the fatty acid treatments. This suggests that reduced cell viability caused by treatment with these siRNAs is truly attributable to depletion of the target gene, and that it is caused by a deficiency in synthesis of fatty acids, as opposed to a buildup of toxic pathway intermediates in the normal culture condition.

To examine the scope of SCD1 involvement in cancer cell survival, several cancer cell lines were subjected to SCD1 or PSMD14 RNAi treatment, in both cases using a single siRNA. Viability of DU145 prostate cancer cells, HCT116 colon cancer cells, and MIA PaCa2 pancreatic cancer cells is reduced (relative to a non-targeting control siRNA) by depletion of both genes as shown in Figure 1C. In all cases, SCD1-depletion-mediated cytotoxicity is rescuable by supplementation of the media with oleate, whereas in all cases PSMD14 depletion is not. This suggests that a variety of cancer cells depend on *synthesis of mono-unsaturated fatty acids* for cell viability, and that SCD1 is a critical node in the pathway that may be a suitable therapeutic target.

The fatty acid synthesis pathway has been studied in the context of both metabolic disease [6] and cancer [7]. Therefore a variety of fatty-acid synthesis inhibitors are available. We set out to use the fatty-acid rescue strategy with several such compounds, as a means of both testing the hypothesis that fatty acid synthesis, and SCD1 activity in particular, are necessary for cancer cell viability, and also with the goal of better understanding the on- and off-mechanism activities of the fatty-acid synthesis inhibitors themselves. As shown in Figure 1D, reference inhibitors for ACC1 (Pfizer #CP640186 [8]), FASN (Merck #10v [9]), and SCD1 (Abbott #7n [10]) all display cytotoxicity and rescue profiles consistent with the pathway position of the target. Toxicity due to ACC1 and FASN inhibition is rescued by palmitate, stearate, and oleate, whereas toxicity due to SCD1 inhibition is rescued only by oleate. It is also noteworthy that the potency of these inhibitors reflects the observation with siRNA. Despite the fact that the reference inhibitors are of comparable potency in biochemical assays on their respective targets, ACC1 and FASN inhibition yield a modest viability reduction, whereas the phenotype with SCD1 inhibition is more pronounced, suggesting the SCD1 is a particularly valuable, perhaps rate-limiting node in this pathway. These observations also suggest that the reference inhibitors are free from dominant (non-rescuable) off-mechanism toxicity in this cell system. The saturated long-chain fatty acids used in the rescue scenario themselves produce a modest viability reduction at the concentrations used. It is noteworthy that these saturated fatty acids are synergistic with the SCD1 inhibitor (Figure 1D). This suggests that while the bulk of the viability impact seen upon SCD1 inhibition is due to depletion of mono-unsaturated fatty acids, SCD1 inhibition also reduces the cells' ability to mitigate the effects of unnatural, exogenous saturated fatty acids, presumably by “detoxification” to oleate or palmitoleate.

### Inhibitor activity clarification by complementation

**Figure 2:**
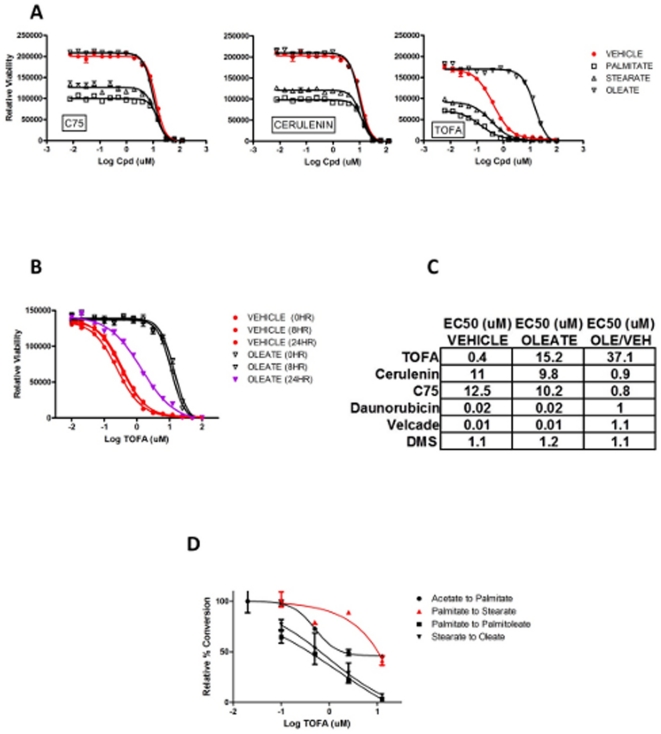
Characterization of various fatty acid synthesis reference inhibitors. **A** HCT116 colon cancer cells were treated with small-molecule FASN inhibitors C75 and Cerulenin (Sigma), or the ACC inhibitor TOFA (Sigma), in media containing fatty acids as indicated, 72 hours prior to viability determination. **B** HCT116 cells were treated with oleate at various times relative to the time of TOFA addition. Cell viability was determined 72 hours after TOFA treatment. **C** HCT116 cells were treated with compounds in media lacking or containing oleate, followed by viability determination after 72 hours. **D** HCT116 colon cancer cells cultured at a density of 1×10^6^ cells per 1 ml media per well in 12-well plates were pre-treated with TOFA for one hour, followed by 13C-Palmitate or 13C-Stearate or 13C-Acetate (Sigma) treatment for four hours. Labeled fatty acids and esters were extracted, saponified, and analyzed by LC/MS/MS using either an API 5000 or API 4000 triple quadrupole mass spectrometer (AB Sciex, Forster City, CA) hyphenated with an Agilent 1100 HPLC system (Agilent, Santa Claire, CA). LC separation was accomplished using Xbridge phenyl 2.1×100 mm column (Waters, Milford, MA). Mobile phase A consisted of 5 mM ammonium formate in deionized water. Mobile phase B consisted of 5 mM ammonium formate in methanol. The sample loading buffer consisted of 30% buffer A and 70% buffer B. A linear gradient was used for the separation (70% to 100% B in 5 min). Samples were ionized by ESI in the negative ion mode and the dwell time for the MRM was 75 ms.

We tested three commercial widely-used historical inhibitors of the fatty-acid synthesis pathway using the “fatty acid rescue” strategy. Cerulenin and C75 are FASN inhibitors [11], [12], and TOFA is an ACC1 inhibitor [13]. As shown in Figure 2A, cerulenin and C75 both inhibit HCT116 colon cancer cell viability as expected. However, neither of these inhibitors is responsive to palmitate, stearate, or oleate, suggesting that both of these inhibitors have dominant, non-mechanism-based cytotoxicity in this cell system, and that reduction in cell viability driven by these compounds is unrelated to inhibition of fatty acid synthesis.

In this assay, TOFA toxicity is rescued effectively by oleate, but not by palmitate or stearate (Figure 2A), contrary to the expectation for a specific inhibitor of ACC1. This pattern of fatty-acid rescue is consistent with TOFA inhibition of SCD1. Alternatively, TOFA-driven cytotoxicity could be altogether off-target (unrescuable by palmitate or stearate), and TOFA could, in principle, physically interact with oleate in the culture medium such that oleate simply prevents TOFA from entering the cells. To test this possibility, we added oleate at various times relative to the time of TOFA addition, and measured viability in all cases at 72 hours after TOFA treatment. As shown in Figure 2B, oleate addition up to 8 hours after TOFA addition yields a degree of “rescue” that is indistinguishable from the case where oleate is added prior to TOFA addition. In this case, TOFA has hours to permeate the cell and inhibit the target, prior to introduction of oleate. This is inconsistent with oleate simply preventing TOFA from entering the cells. When oleate is added 24 hours following TOFA addition, the phenotype reversal is significantly compromised. Therefore, between 8 and 24 hours after TOFA treatment, the cells go past a “point of no return,” and become unresponsive to oleate when assayed for viability at 72 hours.

Additionally we considered that oleate may be a promiscuous “cytotoxicity-rescue-agent.” To test this possibility, we tested a variety of cytotoxic compounds in the oleate-rescue assay. As shown in Figure 2C of a variety of inhibitors of various mechanisms tested (including C75 and cerulenin), only TOFA-driven cytotoxicity is rescuable by oleate. This is inconsistent with oleate acting as a general “rescue-agent.”

To test the hypothesis that the TOFA “fatty acid rescue profile” truly reflects TOFA inhibition of SCD1, we monitored conversion of stable-isotope-labeled fatty acids by LC/MS/MS for fatty-acid flux in the absence or presence of TOFA. We examined the SCD1-mediated desaturation of 13C-palmitate or 13C-stearate to palmitoleate or oleate, respectively, and the elongation of 13C-palmitate to stearate following a 1-hour TOFA exposure. Additionally, to gauge ACC1 inhibition, we monitored 13C-acetate incorporation into palmitate. As shown in Figure 2D, TOFA inhibits both SCD1-mediated desaturation events at a potency comparable to its cell-viability EC50, and comparable to its potency on ACC1. Conversely, substantially higher levels of TOFA are required to effect the (non-SCD1-driven) elongation of palmitate to stearate. Therefore, based on the fatty-acid rescue profile and the direct measurement of SCD1 inhibition in living cells, TOFA inhibits SCD1, which is a previously undescribed activity for TOFA.

In the case of both TOFA and SCD1 inhibitor #7n (Figures 1D and 2A), exogenous palmitate augments the impact of SCD1 inhibition. Beyond the fact that in this assay setting palmitate shows some intrinsic cell viability inhibition, palmitate also left-shifts the EC50 for TOFA and #7n, suggesting that the combination of unnaturally elevated palmitate, and the inability to process it into oleate, enhances viability inhibition of cancer cells.

### Cancer cell viability inhibition tracks with inhibitor potency and SCD1 represents the sole essential desaturation route

**Figure 3:**
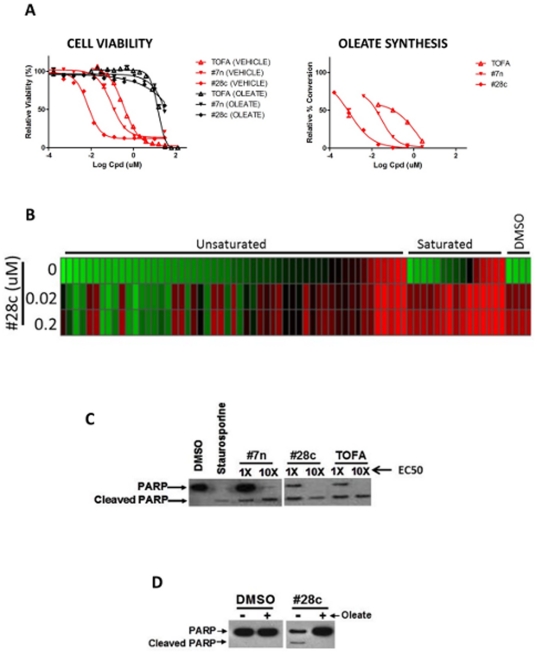
SCD1 inhibitors are potent, specific, and kill cancer cells exclusively by depleting mono-unsaturated fatty acids. **A** HCT116 cells were treated and analyzed for cell viability or cellular SCD1 inhibition (LC/MS/MS) as described above. **B** HCT116 were treated with DMSO or SCD1 inhibitor #28c in the presence of various fatty acids (25 uM) (Biomol, #2803) for 72 hours, and analyzed for cell viability. Data are displayed as a heat map continuum from green (live cells) to red (dead cells). **C** HCT116 cells were treated for 36 hours with various doses of SCD1 inhibitors as indicated. Cells were lysed in LDS loading dye (Invitrogen) and analyzed by western blotting for PARP cleavage (Cell Signaling). Staurosporine, a broad-spectrum kinase inhibitor, was included as a positive control for PARP cleavage. **D** HCT116 cells were treated as in **C**, in the presence or absence of exogenous oleate, followed by analysis of PARP cleavage.

To test the fidelity and correlation of our fatty-acid-rescue viability studies and the cellular SCD1-inhibition LC/MS/MS assay, we examined three SCD1 inhibitor molecules: TOFA, Abbott #7n, and Abbott #28c [14]. As shown in Figure 3A, #7n is several-fold more potent than TOFA, and more oleate-rescuable, in the cell-viability assay, and similarly is more potent than TOFA in the direct cellular SCD1 inhibition assay. Similarly, #28c is substantially more potent than #7n in the direct cellular SCD1 inhibition assay, and also in the cell viability assay, while completely retaining oleate-rescuability.

We considered that other unsaturation events may be available to support cancer cell viability, via desaturases other than SCD1, if cells are given sufficient supply of saturated substrate. To explore this possibility, as well as to further characterize the specificity of the #28c SCD1 inhibitor we examined a panel of fatty acids of varying chain lengths and saturation states for their ability to “complement” (prevent the effect of) SCD1-inhibitor-mediated viability reduction. As shown in Figure 3B, while not all unsaturated fatty acids complemented, all “complementing” fatty acids did contain at least one unsaturated bond. On the other hand, all saturated fatty acids failed to complement, suggesting that unsaturation is absolutely required for complementation, and that alternative desaturase activities cannot be employed.

We examined TOFA, #7n, and #28c for induction of the apoptosis cascade. HCT116 cells were treated with compound doses equivalent to, or ten-fold above, their cytotoxocity IC50. As shown in Figure 3C, all three fatty-acid synthesis inhibitors induce PARP cleavage in a dose-dependent fashion, suggesting that interruption of fatty-acid synthesis in these cells causes apoptosis. The induction of PARP cleavage correlates with cell death in that it is reversible with exogenous oleate (Figure 3D). PARP cleavage is a marker of apoptosis [15].

### SCD1 inhibition slows tumor growth and is not universally toxic

**Figure 4:**
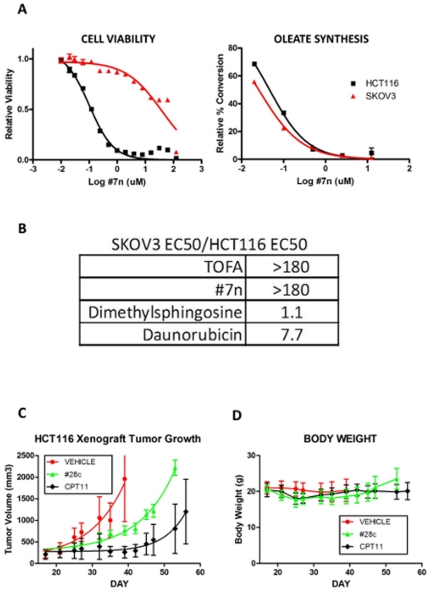
SCD1 inhibition is not universally toxic, and slows growth of HCT116 xenograft tumors. **A** HCT116 or SKOV3 cells were treated and analyzed for cell viability or cellular SCD1 inhibition (LC/MS/MS) as described above. **B** HCT116 or SKOV3 cells were treated and analyzed for cell viability. Table expresses the ratio of SKOV3 EC50 versus HCT116 EC50. **C**, **D** Nude mice harboring passage five 200 mm3 HCT116 tumors (passaged as trocar fragments) (n = 10 per group) were dosed by oral gavage twice daily with 160 mg/kg #28c for 20 days or with intravenous CPT11 on three consecutive days starting when tumors reached 200 mm3. Tumor growth (**C**) and body weight (**D**) were monitored and plotted as mean +/− standard deviation.

The impact of SCD1 depletion on several cancer cell lines raises the possibility that SCD1 inhibition will be universally toxic. We tested the ovarian cancer cell line SKOV3 for sensitivity to a reference SCD1 inhibitor. As shown in Figure 4A, the SCD1 inhibitor has limited impact on SKOV3 cell viability, versus HCT116, despite having comparable inhibitory effect on cellular conversion of stearate to oleate (SCD1 activity). We considered that SKOV3 may be generally insensitive to various toxic agents. As shown in Figure 4B, SKOV3 and HCT116 are comparably sensitive to a variety of mechanistically-distinct toxic compounds, such as dimethylsphingosine and daunorubicin, whereas in the case of SCD1 inhibitors, SKOV3 is quite insensitive relative to HCT116. This suggests that SKOV3 has some specific insensitivity to fatty-acid-synthesis inhibition, and that SCD1 inhibition will not be universally toxic.

SCD1 inhibitor #28c was recently described by Abbott [14] as a potent, orally-available SCD1 inhibitor with favorable pharmacokinetic properties. Therefore this molecule provides a tool for *in vivo* SCD1 target validation for cancer. We treated mice bearing 200 mm3 HCT116 tumors twice daily with #28c by oral gavage (versus IV CPT11 on an optimized dosing regimen) for 20 days and monitored tumor growth and body weight. As in Figure 4C, #28c showed moderate growth delay of HCT116 tumors. While the SCD1 inhibitor did reduce the oleate content of excised tumors (not shown), substantial oleate remained in the tumor tissue, raising the possibility that dietary oleate may be limiting for efficacy of the SCD1 inhibitor. Treatment with the SCD1 inhibitor was accompanied by weight loss approaching 20%, or reduction from 20 g to approximately 16 g, on dosing day 10 (study day 26) (Figure 4D) that was recovered after cessation of dosing (study day 36). It is unclear whether this weight loss is on-mechanism for this inhibitor (as might be expected from an inhibitor of lipid synthesis), or unrelated to SCD1 inhibition.

## Discussion

Cancer cells are distinct from nonmalignant cells based partly on their unique metabolic status , one element of which is an unusual requirement for fatty acid synthesis [16]. Thus the fatty acid synthesis pathway has been an attractive cancer target for some time, and primary attention has focused on fatty acid synthase, which marks the point of production of long-chain fatty acids [17]. Our experiments and others [18] suggest, however, that the rate-determining step in synthesis of mono-unsaturated fatty acids is at the point of de-saturation (by SCD1), and that SCD1 represents a particularly vulnerable node in this pathway. Using both siRNA and reference inhibitors, we have shown that loss of SCD1 activity yields pronounced viability inhibition of various cancer cells *in vitro*. The fact that this viability inhibition is reversible when oleate is added to the cell culture medium argues that the phenotype is on-mechanism, and attributable to an SCD1-inhibition-mediated oleate deficiency, as opposed to the buildup of intracellular palmitate or other upstream pathway components. We propose that this fatty-acid rescue strategy is a simple, broadly useful mechanism for characterization of fatty acid synthesis inhibitor specificity, as evidenced by our characterization of the SCD1 inhibitory activity of TOFA.

HCT116 tumor growth is delayed upon treatment with a potent, orally-available SCD1 reference inhibitor. However, tumor growth delay is moderate, falling considerably short of that seen with CPT11, which served as a positive control. It is noteworthy, though, that an optimized dosing regimen was not established for the SCD1 inhibitor. Tumor growth delay was accompanied by weight loss, and decreased body weight was sustained throughout the course of dosing. SCD1 was originally investigated as a target for metabolic disorders, and it would not be surprising if some portion of the observed weight loss was due to broad inhibition of *mono-unsaturated* fatty acid synthesis. Additionally, while SCD1 knockout mice are viable [19], mice have several SCD isoforms, which may be redundant. If the toxicity (weight loss) seen in the current study is due to fatty acid synthesis inhibition, this may be attributable to the SCD1 inhibitor targeting multiple murine SCD isoforms. This was not tested. Nevertheless, in principle this would differentiate the inhibitor-driven toxicity profile from the genetic SCD1 knockout. Alternatively, the weight loss seen could be the net effect of SCD1-inhibition-driven reduced adiposity and increased energy expenditure, comparable to that seen in the SCD1 knockout [19], [20].

Mono-unsaturated fatty acid maturation and processing, following production by SCD1, is a complex network leading to a host of different chain lengths, saturation states, and subcellular distribution fates. It may be that while SCD1 represents a final, rate-limiting “point-of-constriction” in the pathway, a downstream enzyme target, along one of a variety of mono-unsaturated fatty-acid processing sub-pathways, may represent a node that is specifically required for cancer cell viability, and dispensable for normal cell function. It may also be the case that SCD1 inhibition could be productive in a co-treatment scenario, at low doses in conjunction with a traditional agent.

## Materials and Methods

### Cell Culture

HCT116, DU145, and MIA PaCa2 cancer cells were obtained from ATCC and maintained in RPMI1640 (Cambrex) supplemented with penn-strep (ATCC) and 5% FBS (Hyclone). For assays, cells were plated in RPMI1640 lacking penn-strep and containing 2% FBS at a density of 4000 cells/100 ul/well in 96 well plates for siRNA treatment and viability determination, or at a density of 1000 cells/25 ul/well in 384 well plates for compound treatment and viability determination, or a density of 1×10^6^ cells/1 ml/well in 12-well plates for compound treatment and LC/MS/MS analysis of labeled fatty-acid flux. All cells were grown as a monolayer in 95% air/5% CO_2_, and a single lot of non-delipidated FBS was used for all experiments

### Fatty Acid Preparation

Fatty acids (Sigma) were dissolved in methanol to a concentration of 25 mM. 25 mM stocks were then diluted ten-fold in PBS (Cambrex) containing 0.9% BSA (A9576, Sigma). These 2.5 mM (100X) stocks were thoroughly mixed and incubated in a 37 degree water bath for one hour prior to aliquoting and freezing at −20 degrees. The fatty acid panel from Biomol (#2803) was dissolved in DMSO.

### LC/MS/MS Analysis

All experiments were performed by using a API 5000 or API 4000 triple quadrupole mass spectrometer (AB/MDS Sciex, Concord, Canada) and an Agilent 1100 HPLC pump (Agilent, Andover, MA). Columns were Xbridge phenyl 2.1×100 mm (Waters, Milford, MA). Buffer A was water with 5 mM ammonium formate; buffer B was methanol 5 mM ammonium formate; and loading buffer was 30% buffer A plus 70% buffer B). A 5-min gradient (70% to 100%buffer B, linear) was used with MRM acquisition time of 75 msec using negative mode. Fatty acid synthesis determinations were expressed either as product/(product+substrate) (in the case of stearate, palmitoleate, and oleate synthesis), or as raw product normalized to unlabeled linoleic acid (in the case of palmitate synthesis).

### siRNA transfection

ON-Target-PLUS or siGENOME stock siRNAs (gene-specific or non-targeting controls) were purchased from Dharmacon, and transfected (50 nM (12.5 nM per each of four siRNAs) in the case of siRNA pools (Smartpools), or 25 nM in the case of single siRNAs using Lipofectamine 2000. The SCD1 single siRNA sequence was: 5′-GAACAGUGCUGCCCACCUC-3′. The PSMD14 single siRNA sequence was: 5′-GGCAUUAAUUCAUGGACUA-3′. Sixteen hours after transfection, 1/100^th^ volume of 100× BSA-complexed fatty acid (palmitate, stearate, or oleate) (100X = 2.5 mM) or vehicle alone was added. Seventy-two hours after transfection, 25 ul Cell-titer Glo was added and plates were analyzed for cell viability according to the manufacturer (Promega) recommendation. All analyses were performed in duplicate or triplicate, on multiple occasions with similar outcomes, and are graphed as the mean ± SD of a single representative experiment.

### Small-molecule inhibitor treatment

C75, cerulenin, and TOFA were purchased from Sigma. ACC inhibitor CP640186 (Pfizer), FASN inhibitor #10v (Merck), and SCD1 inhibitors #7n and #28c (Abbott) were synthesized at Genzyme (Waltham, MA). Compounds were dissolved in DMSO and stored at −20 degrees. In the case of both BSA-complexed fatty acid (or vehicle) and small-molecule inhibitors (or DMSO), agents were pre-diluted in assay culture medium to 7× final concentration. 5 ul apiece of 7× stocks of two appropriate agents was added to 25 ul medium, for 35 ul final volume. 72 to 96 hours later, 7.3 ul Cell-titer Glo was added, and plates were analyzed for cell viability. All analyses were performed in duplicate or triplicate, on multiple occasions with similar outcomes, and are graphed as the mean ± SEM of a single representative experiment.

### HCT116 Xenograft

Animal studies (#GENZ100507-20) were performed following approval of Genzyme Institutional Animal Care and Use Committee (IACUC). HCT116 tumors were passaged as trocar fragments in *nu/nu* mice. Animals bearing passage five tumors were treated with #28c (160 mg/kg twice daily oral dosing for 20 days) or CPT11 (once daily intravenous dosing for three consecutive days) when tumors reached 200 mm3.
